# Weather-dependent changes in habitat use by Alpine chamois

**DOI:** 10.1186/s40462-024-00449-x

**Published:** 2024-01-16

**Authors:** Pia Anderwald, Sven Buchmann, Thomas Rempfler, Flurin Filli

**Affiliations:** Swiss National Park, Chastè Planta-Wildenberg, Runatsch 124, 7530 Zernez, Switzerland

**Keywords:** Climate change, Protected area, *Rupicapra rupicapra*, Step selection functions, iSSF, Ungulate

## Abstract

**Background:**

Alterations in weather patterns due to climate change are accelerated in alpine environments, but mountains also provide a wide range of niches and potential refuge areas. In order to identify future critical habitat for mountain ungulates for effective protection, it is important to understand their spatial responses to changing weather conditions without movement constraints by human disturbance.

**Methods:**

Using integrated step selection functions, we investigated fine-scale changes in seasonal habitat use in response to weather and time of day for 55 GPS-collared adult Alpine chamois in summer and 42 individuals in winter in a strictly protected area.

**Results:**

Chamois reacted to increasing precipitation and wind speeds primarily by moving to lower elevations in summer and winter. However, reactions to high summer temperatures predominantly involved preferences for increasing tree cover density and northerly slopes. Snow depth had little effect on habitat choice, and southerly slopes were preferred in winter regardless of temperature. At night, chamois moved to steeper slopes and lower elevations than during daytime in both seasons, and to more open areas in summer. Steeper slopes were also preferred with increasing tree cover density.

**Conclusions:**

Chamois employ adaptive fine-scale adjustments in their habitat choice consistent with respect to efficient thermoregulation and protection from both weather extremes and predation risk in summer and winter. Movement responses to climate change are therefore expected to be far more complex than simple altitudinal changes in distribution. Particularly the role of forest cover must not be underestimated, as it appears to provide important thermal refuge habitat from high summer temperatures.

**Supplementary Information:**

The online version contains supplementary material available at 10.1186/s40462-024-00449-x.

## Background

Limitations for suitable habitats of a species are determined more by extreme rather than average environmental conditions [13, 40, 78]. Weather extremes, particularly with respect to temperature and precipitation patterns, are being shifted and becoming more frequent under the influence of climate change and are likely to alter species’ distributions unless they can find refuges within their current ranges [24, 28, 51]. Individual responses to different weather conditions can give first indications of such possible refuges from future climate change, as the fastest way for animals to respond to unfavourable weather or climate is through behavioural adaptations (e.g. [21]). Larger mammals with their slow life histories are more affected by climate change than smaller species, as they typically also have more difficulties in finding suitable microclimates [35, 49]. However, provided that individuals’ home ranges are sufficiently heterogeneous, temporal refuges may be available at fine spatial scales even for large mammals.

Mountain regions represent the spatially most diverse environments on earth, accommodating a high variety of climatic niches at fine local scales. On the other hand, they are also predicted to face amongst the most rapid and pronounced changes in weather conditions linked to anthropogenically caused climate change in the near future, including new temperature and precipitation extremes and decreases in snow cover [31, 36, 73]. Accelerated temperature changes in alpine habitats have already caused range shifts in numerous species [25, 75]. Amongst the most mobile mammals at high elevations are mountain ungulates which are suspected to be at risk from climate change due to the possibility of phenological mismatches with forage plants [45, 56], weight loss through reduced diurnal foraging activity due to the risk of overheating in summer [47] and increased competition with other ungulates [2, 3, 32, 33, 44]. The short vegetation period at high elevations is likely to exacerbate fitness trade-offs typically faced by herbivores such as maximizing energy gain vs. minimizing predation risk [76] or seeking shelter [53, 64]. This forces mountain ungulates to balance several needs simultaneously in their habitat selection [12]. Although recent upward shifts in distributional ranges have been reported for this group [22], determining causes is difficult due to complex intra- and interspecific interactions. For example, Alpine ibex (*Capra ibex*) move to higher elevations during hot summer days, and this applies particularly to males which have a lower surface to volume ratio and therefore more difficulties in dissipating heat than females [10]. However, males also move upslope at higher population density, and both sexes increase their use of higher elevations at high red deer (*Cervus elaphus*) densities, suggesting additive effects of weather, intra- and interspecific competition [41]. Similarly, Mason et al. [48] reported a stronger upward shift in chamois (*Rupicapra rupicapra*) distribution in response to the presence of domestic sheep (*Ovis aries*) than to higher temperatures.

Although temporarily seeking refuge areas may mitigate against direct negative effects from changing weather patterns, such movements are often constrained by human infrastructure or activities [1, 72], and refuge areas may offer less optimal feeding opportunities. For example, forests can play an important role as refuges from inclement weather, as they buffer against temperature extremes and provide protection from high winds or rainfall [30]. Accordingly, they are also used by ungulates as shelter from storms [26] or heat [17, 74]. However, forage quantity is often reduced in the forest compared to open areas that receive more sunlight [43, 64], representing a potential trade-off between foraging efficiency and cover. Understanding changes in habitat choice with altering weather conditions based on long-term studies with sufficient variability in environmental parameters, and ideally in the absence of constraints imposed by humans, can help discern priorities to the animals and predict future habitat requirements.

In order to investigate how a relatively generalist mountain ungulate species seasonally adjusts its fine-scale habitat use to changing weather patterns under conditions largely unconstrained by humans, we analysed positions of GPS-collared Alpine chamois (*Rupicapra rupicapra*) over a 14-year period in an area strictly protected from human activities such as hunting and lifestock grazing. Like other mountain ungulates, chamois are well adapted to topographically complex landscapes and use steep slopes as escape terrain from predators. They most commonly occur on alpine grasslands and in conifer forests. While some populations undertake seasonal altitudinal movements between the two habitat types, others remain in the forest year-round [27]. We hypothesized that besides foraging opportunities, important drivers of fine-scale habitat selection in chamois were efficient thermoregulation, potential shelter at times of inclement weather, and safety from predators [53]. This led to the following predictions:

1. Thermoregulation: Endotherm animals accomplish a more favourable energy balance by selecting environments with temperatures close to their species- and season-specific thermoneutral zones [60]. Chamois should thus avoid temperature extremes both in summer and winter. Particularly in cold winters, this could be achieved by moving to lower elevations and into the protection of the forest, and by selecting more southerly and easterly slopes with increased duration of sunlight. At high temperatures in summer, the opposite would be expected, with individuals selecting northerly slopes and higher elevations [10, 22] to take advantage of more exposure to the wind along ridges. However, alternatively, they could retreat to the relative shade and cooler temperatures of the forest [17, 74].
2. Shelter: Chamois should prefer more forested areas at lower elevations to open areas at high elevations under conditions of heavy precipitation and at high wind speeds to seek shelter [26]. The same applies to periods of deep snow cover in winter. However, during benign weather conditions in summer, open areas at high elevations are likely to be selected due to better foraging opportunities [5].
3. Safety: Chamois rely predominantly on steep slopes as safety habitat [77]. Steep slopes should therefore be sought by the animals particularly under circumstances of poor visual or acoustic predator detection capability, i.e. at night or at times of strong precipitation or high wind speeds.

## Methods

### Study area

**Fig. 1:**
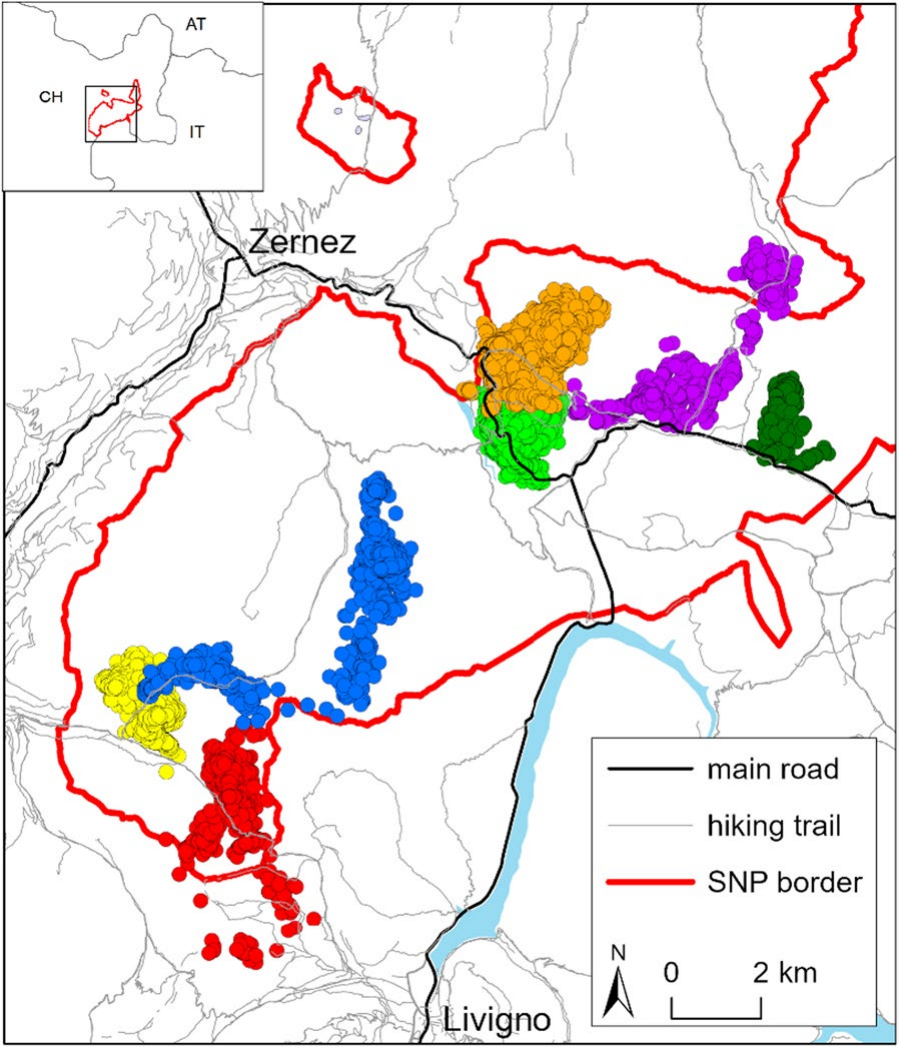
Overview of the study area in eastern Switzerland (inset: CH = Switzerland, AT = Austria, IT = Italy) with the Swiss National Park and locations of 7 individual chamois (out of 57 collared within the park) to give an indication of home range sizes. Light blue areas represent lakes. Map: Data: swisstopo, OpenStreetMap, SNP © SNP 2024/10

The study was conducted in the Swiss National Park (SNP), the oldest (founded in 1914) and most strictly protected area in central Europe (IUCN category Ia). Located in eastern Switzerland in the central Alps (46.65° N, 10.17° E; Fig. 1), it extends over an area of 170 km^2^ and comprises elevations between 1380 and 3173 m asl. About 30% of the area consist of conifer forest (dominated for the most part by mountain pine *Pinus mugo*, with some larch *Larix decidua*, cembra pine *Pinus cembra*, Norway spruce *Picea abies* and Scots pine *Pinus sylvestris*), 20% of grassland, and 50% of unvegetated ground (mainly rock and scree) [37, 80]. Weather conditions are typical of a continental dry inner-alpine climate. Over the duration of the study (April 2008–March 2022), air temperatures ranged from an average annual minimum of − 27.8 ± 2.9 °C to a maximum of 25.2 ± 1.3 °C, and annual precipitation ranged from 592 to 1032 mm (based on data recorded at the weather station Buffalora at 1971 m asl.). Maximum annual snow cover ranged from 50 to 138 cm at the weather station Samedan at 1750 m asl.; [50]).

There is no hunting of wildlife within the park boundaries at any time of year, and supplementary feeding is prohibited both in the park and elsewhere in the canton of Grisons. Only one (cantonal) road runs through the SNP; the only other access is by a network of 100 km of hiking trails. Visitors are fined for leaving the trails or bringing dogs into the park. Due to the danger of avalanches and to avoid disturbance to wildlife, the entire SNP is closed to visitors during winter (ca. mid November to the end of May). Over the study period, the only large mammalian predators present in the area were single vagrant brown bears (*Ursus arctos*) in spring or summer and a single resident wolf (*Canis lupus*) since December 2016. On the other hand, golden eagles (*Aquila chrysaetos*) and red foxes (*Vulpes vulpes*) were present throughout and represented a danger to chamois kids.

### Telemetry data

A total of 57 chamois (34 adult females and 23 adult males) were fitted with GPS Plus or Vertex Plus (the latter from 2020 onward) collars (VECTRONIC Aerospace GmbH, Berlin, Germany) and coloured/numbered ear tags for individual recognition between 2008 and 2021. Collars were programmed to take a GPS position every 4 h. Animals were either captured in box traps or tranquilized in the field with 0.3–0.8 ml Hellabrunner mixture (125 mg Xylazin & 100 mg Ketamin/ml) administered by air rifle from a distance of up to 30 m. After 1 h, they were antagonized with 0.3–0.8 ml Atipamezole and set free. Throughout the deployment, data were downloaded via UHF (Ultra High Frequency) approximately once per month. Depending on battery performance, the drop-off was activated via UHF and the collar retrieved after 1.5 to 2 years. All animal handling was carried out in accordance with Swiss animal welfare laws and under permit from the cantonal and federal authorities (permit numbers 1/2008, 2011_07, 2014_07F, 2017_12F, GR 2020_08F, GR/01/2021).

### Habitat and weather variables

Topographic habitat variables were extracted from a digital elevation model of 4 m × 4 m resolution, based on digital photogrammetry [38] and included elevation (metres above sea level), slope (degrees) and aspect (degrees). The latter two parameters were calculated from the digital elevation model using the Surface tool of the Spatial Analyst Extension in ArcGIS Desktop 10.7.1. In order to avoid including a circular variable in the habitat models, aspect was split into the two linear variables northness (calculated as cos (aspect * π /180)) and eastness (sin (aspect * π /180)), with values of − 1 representing southern and western, and 1 representing northern and eastern slopes, respectively [79]. Tree cover density (percent) was extracted from the Tree Cover Density datasets for 2012, 2015 (both 20 m resolution) and 2018 (10 m resolution) of the Copernicus Land Monitoring Service [42]. The 2018 dataset was resampled to 20 m resolution (Additional file 1: Fig. A1).

Weather data for the analysis included hourly average temperature (degrees Celsius) and maximum wind speed (kilometres per hour), and precipitation (millimetres) summed over 3 h. These parameters were obtained from the weather station Buffalora at 1971 m asl., located ca. 40 m outside the park’s eastern boundary [50]. Due to gaps in snow cover data at this weather station, snow depth (centimetres; measured at 5:00 each morning) was obtained from the station Samedan at 1750 m asl., located 15 km from the southwestern boundary of the SNP [50].

### Statistical analysis

All analyses were conducted in R version 4.1.3 [57]. Following deletion of inaccurate animal positions (n = 29; [15]), 10 random steps were generated for each realized step (thus representing strata of 11 positions) based on a gamma distribution for step lengths and a uniform distribution for turning angles for each individual using the R package ‘amt’ [68, 69]. Both actual and available positions were then linked spatially with their corresponding habitat variables and temporally with the weather variables from the weather station. For tree cover density, the value closest in time to the corresponding available dataset (2012, 2015 or 2018) was extracted for each position. All timestamps were assigned to day- or nighttime based on local sunrise (defined as the time when the top edge of the sun appears on the horizon) and sunset (sun disappears below the horizon) using the package ‘suncalc’ [71]. Seasons were defined as summer (June to October) and winter (i.e. snow covered; December to April). November and May were excluded from the analysis, as the extent of snow cover during these ‘transition months’ varied greatly between years. Individuals were only considered for the analysis if sample sizes reached at least 80% of theoretically possible positions over the 5 months of summer or winter, respectively. This resulted in sample sizes of 55 adult individuals (32 females, 23 males) for summer and 42 individuals (25 females, 17 males) for winter. The analysis described below was first conducted for males and females separately, but as no major differences were detected, both sexes were pooled.

In order to investigate how chamois adjusted their habitat use to changing weather conditions, integrated step selection functions (iSSF; [11] were applied to the summer and winter data separately. Two generalized linear mixed effects models with a Poisson distribution were fitted to used and available end positions of each step using the glmmTMB package [19]. While individual-specific random slopes were included for each habitat variable, the intercept was estimated per stratum, with a variance fixed at 10^6^ in order to avoid shrinkage of intercepts [52]. Step length was included in the models as a fixed effect to account for potential biases in selection estimates [34]. First-order interaction terms between habitat and weather variables (all continuous, and day-/nighttime, respectively, were included in the full models where they made sense biologically. Interactions with all weather variables, as well as day-/nighttime, were thus included for elevation and tree cover density, interactions with slope were included for precipitation, wind speed, snow cover and day-/nighttime, but not for temperature; the only interaction term for northness was included with temperature, while eastness occurred in no interaction. Interactions between habitat variables were restricted to tree cover density * slope. Besides the inclusion of snow depth in winter, summer and winter models were identical. All continuous explanatory variables were centred on the mean and divided by the standard deviation in order to enable direct comparisons of effect sizes and to avoid convergence problems. The strongest correlations between explanatory variables were detected between elevation and tree cover density for the summer (Pearson’s r = − 0.616) and winter (Pearson’s r = − 0.469) datasets, respectively, and between wind and temperature in summer (Pearson’s r = 0.481) and winter (Pearson’s r = 0.403; Additional file 1: Table A1). These correlations were not considered to preclude interpretation of the model results, and all variables were therefore included. Model selection was performed in a stepwise backward manner based on AIC [23]: in the first step, each interaction term (or single variable where possible) was removed from the full model in turn and all models compared. The model with the lowest AIC was selected. The process was then repeated comparing all possible models at each step until no more removal of an interaction or variable led to a further decrease in AIC. The R code for the graphical representations of model results was adapted from Sigrist et al. [70].

## Results

The best supported summer model (ΔAIC = 3.5 relative to the full model) contained all interaction terms from the full model except precipitation with slope, precipitation with tree cover density, and wind with slope (Table 1). The model that included precipitation with slope only differed by ΔAIC =  + 0.9, but was also less parsimonious.

**Table 1:** Results of the final generalized linear mixed effects models for chamois habitat use in summer (June to October) and winter (December to April) in the SNP

Predictor	Summer		Winter	
	Estimate (SE)	95% CI	Estimate (SE)	95% CI
Step length	0.208 (0.003)***	0.201, 0.214	− 0.294 (0.007)***	− 0.307, − 0.281
Elevation	− 0.051 (0.058)	− 0.165, 0.063	− 0.370 (0.070)***	− 0.507, − 0.234
Slope	− 0.044 (0.015)**	− 0.073, − 0.015	− 0.001 (0.030)	− 0.060, 0.057
Tree cover density (tcd)	0.0003 (0.011)	− 0.021, 0.022	− 0.033 (0.024)	− 0.080, 0.015
Northness	− 0.083 (0.040)*	− 0.161, − 0.004	− 0.255 (0.055)***	− 0.363, − 0.147
Eastness	− 0.063 (0.033)	− 0.128, 0.002	0.121 (0.044)**	0.035, 0.206
Precipitation:elevation	− 0.140 (0.012)***	− 0.163, − 0.117	− 0.114 (0.018)***	− 0.150, − 0.079
Precipitation:slope	−	−	0.017 (0.006)**	0.005, 0.028
Precipitation:tcd	−	−	0.030 (0.007)***	0.017, 0.043
Temperature:elevation	0.055 (0.014)***	0.028, 0.082	− 0.085 (0.018)***	− 0.121, − 0.050
Temperature:tcd	0.032 (0.008)***	0.016, 0.048	− 0.022 (0.008)**	− 0.038, − 0.007
Temperature:northness	0.076 (0.009)***	0.059, 0.093	−	−
Wind:elevation	− 0.142 (0.014)***	− 0.169, − 0.115	− 0.244 (0.018)***	− 0.279, − 0.209
Wind:slope	−	−	−	−
Wind:tcd	0.021 (0.008)*	0.005, 0.036	0.025 (0.007)***	0.010, 0.039
Snow:elevation	n.a	n.a	0.037 (0.018)*	0.002, 0.072
Snow:slope	n.a	n.a	−	−
Snow:tcd	n.a	n.a	0.022 (0.008)**	0.006, 0.038
Day/night:elevation	− 0.310 (0.027)***	− 0.363, − 0.258	− 0.089 (0.034)**	− 0.157, − 0.022
Day/night:slope	0.177 (0.010)***	0.157, 0.198	0.156 (0.012)***	0.132, 0.179
Day/night:tcd	− 0.124 (0.016)***	− 0.155, − 0.094	−	−
tcd:slope	0.078 (0.006)***	0.067, 0.090	0.059 (0.007)***	0.044, 0.073
N_individuals_	55		42	
N_strata_	75,195		54,898	

Coefficients correspond to the scaled variables used in the models. − indicates that the interaction was included in the full model, but was removed in the step-wise backward selection according to AIC; n.a. indicates that the parameter was not included in the model from the start. SE = standard error, CI = confidence interval

*significant at *p* = 0.05, **significant *p* = 0.01, ***significant at *p* = 0.001

The best supported winter model (ΔAIC = 3.4 relative to the full model) contained four interaction terms fewer than the full model (temperature with northness, wind with slope, snow with slope, and day/night with tree cover density; Table 1). The second best model for winter, including day/night with tree cover density, only differed by ΔAIC =  + 0.2, but was also less parsimonious than the best supported model.

During summer, chamois in the SNP travelled longer distances between consecutive GPS locations (median = 128 m; 25% percentile = 55 m, 75% percentile = 263 m;) compared to randomly generated step lengths, while travel distances were significantly shorter than random steps in winter (median = 63 m; 25% percentile = 21 m, 75% percentile = 147 m; Table 1).

**Fig. 2:**
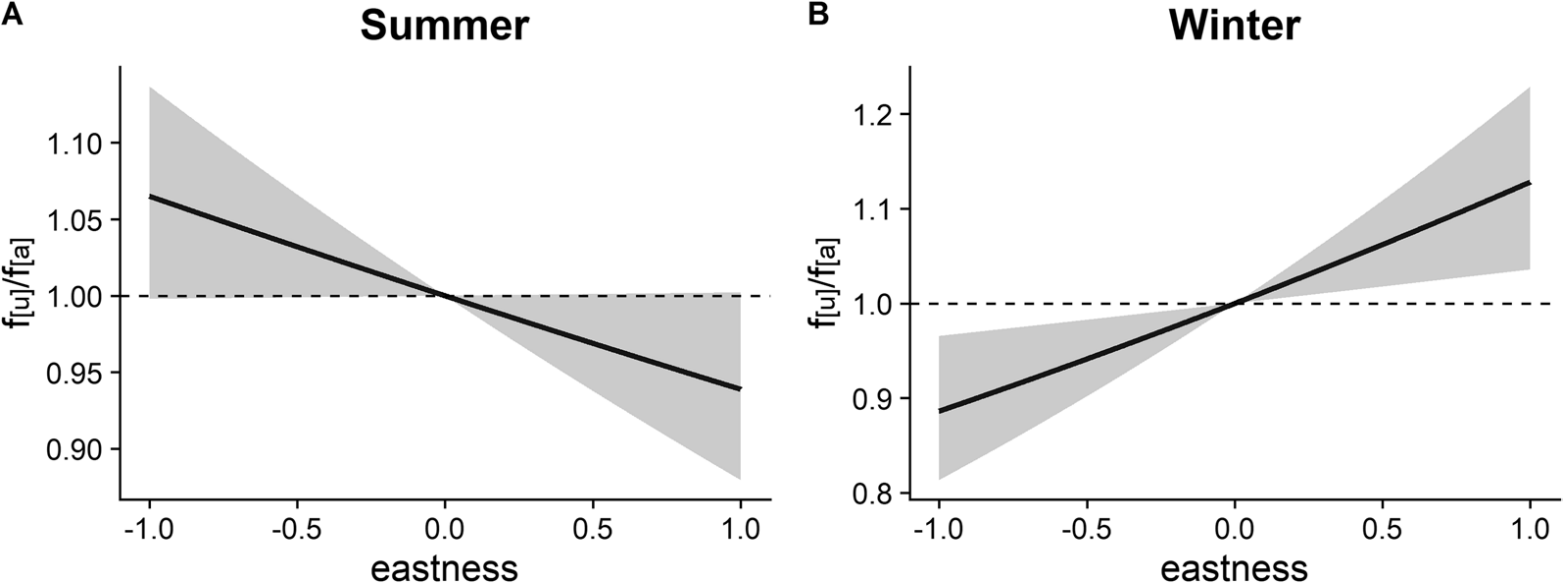
Habitat choice of chamois with respect to eastness (with 95% confidence intervals) in **A** summer and **B** winter. f_[u]_/f_[a]_ represents the frequency ratio between used and available positions, with values > 1 indicating preference and values < 1 avoidance. Note the different scales of the y-axis between **A** and **B**

Eastness, the only habitat variable that was not included in an interaction term, only had a marginal negative effect on habitat choice by chamois in summer, but easterly slopes were selected for in winter (Table 1, Fig. 2).

**Fig. 3:**
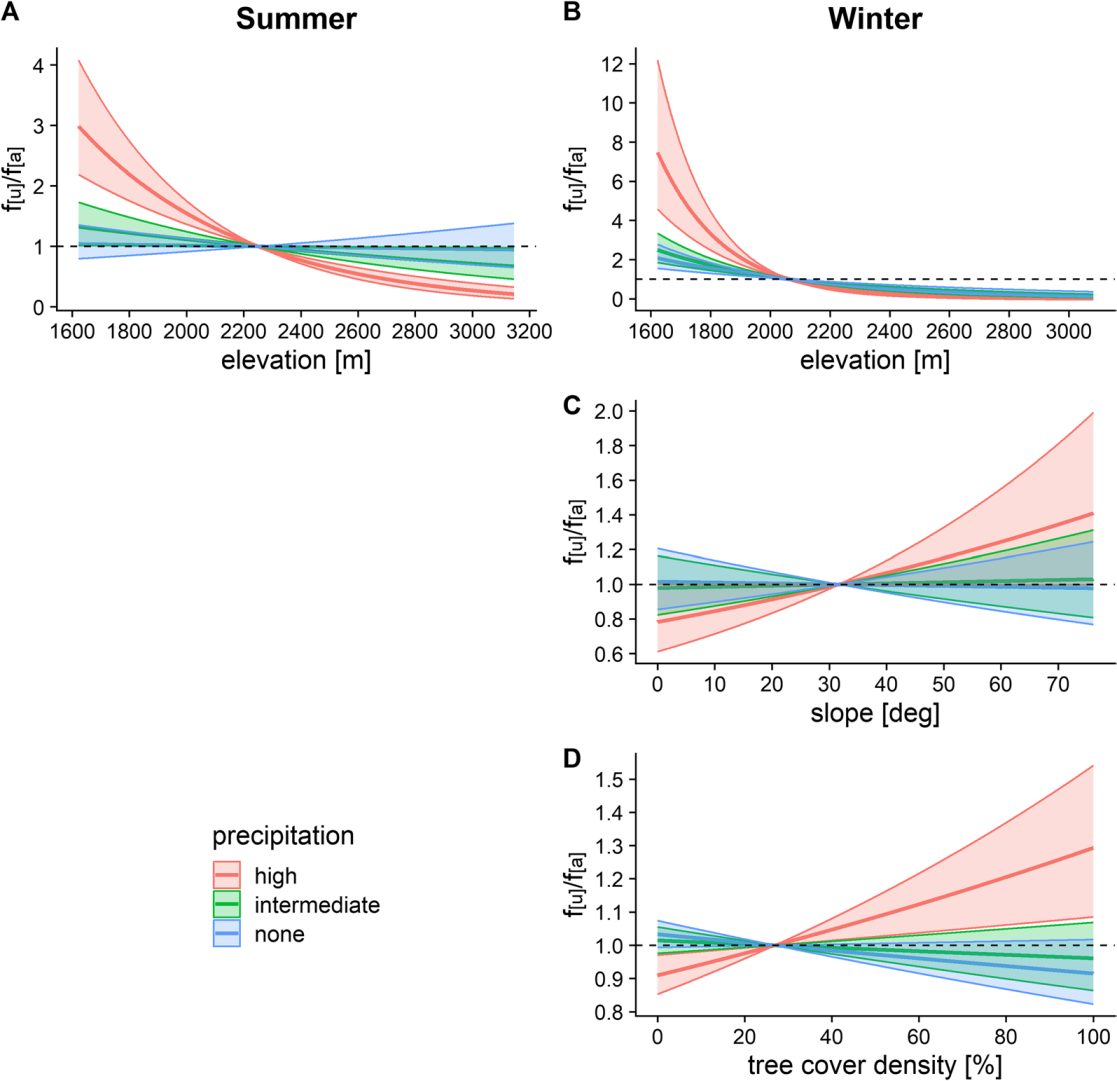
Effects of precipitation on chamois habitat choice with respect to elevation in summer (**A**) and winter (**B**), and slope (**C**) and tree cover density (**D**) in winter. Interactions between precipitation and slope and tree cover density for summer were not included in the final model according to AIC. Precipitation values were skewed towards 0; for the plot, intermediate and high precipitation were therefore set to 1.1 mm and 5 mm in summer (maximum = 23.1 mm in the data), and 0.5 mm and 3.8 mm in winter (maximum = 16.5 mm in the data), respectively, determined visually from the density distribution of precipitation values. f_[u]_/f_[a]_ represents the frequency ratio between used and available positions, with values > 1 indicating preference and values < 1 avoidance. Note the different scales of the y-axis between graphs

Although chamois selected for lower elevations in any weather conditions during winter, this was exacerbated at high levels of precipitation in both seasons (Fig. 3A, B), while no elevational selection was detected at intermediate or no precipitation during summer (Fig. 3A). Compared to its effects on the choice of elevation, precipitation only had a weak influence on the selection of slope and tree cover density: no effects were detected for interactions with either habitat variable in summer, while chamois selected for marginally steeper slopes and higher tree cover densities at high levels of precipitation in winter (Table 1; Fig. 3C, D).

**Fig. 4:**
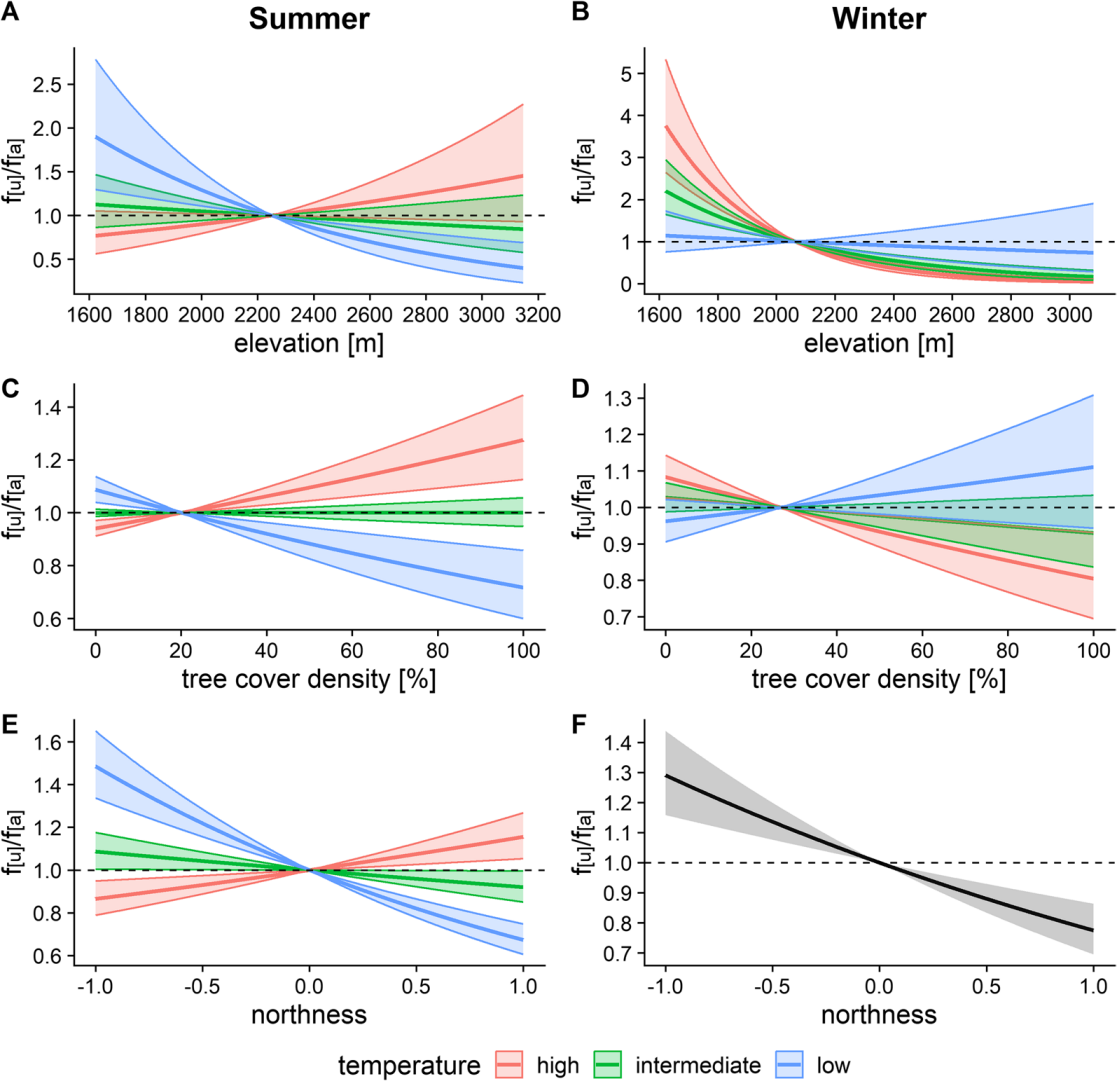
Effects of temperature on chamois habitat choice with respect to elevation (**A**–**B**), tree cover density (**C**–**D**) and northness (**E**–**F**) in summer (left panel) and winter (right panel). Low, intermediate and high temperatures correspond to seasonal minimum, mean and maximum values. f_[u]_/f_[a]_ represents the frequency ratio between used and available positions, with values > 1 indicating preference and values < 1 avoidance. Note the different scales of the y-axis between graphs

The effects of temperature on chamois habitat use were reversed between summer and winter. While the animals showed a preference for lower elevations at low temperatures in summer with no altitudinal selection at intermediate or high temperatures (Fig. 4A), they increasingly preferred lower elevations with increasing temperatures in winter (Fig. 4B). Instead of a selection for elevation, chamois preferred areas with denser tree cover at high temperatures in summer, while selecting for open habitat at low temperatures (Fig. 4C). In winter, temperature-dependent selection for tree cover density was less pronounced, but the animals showed a weak avoidance of denser tree cover at high temperatures (Fig. 4D). Northerly slopes were positively selected for at high temperatures in summer, with no selection at intermediate, and negative selection at low summer temperatures (Table 1, Fig. 4E), but were clearly avoided in winter independent of temperature (Fig. 4F).

**Fig. 5:**
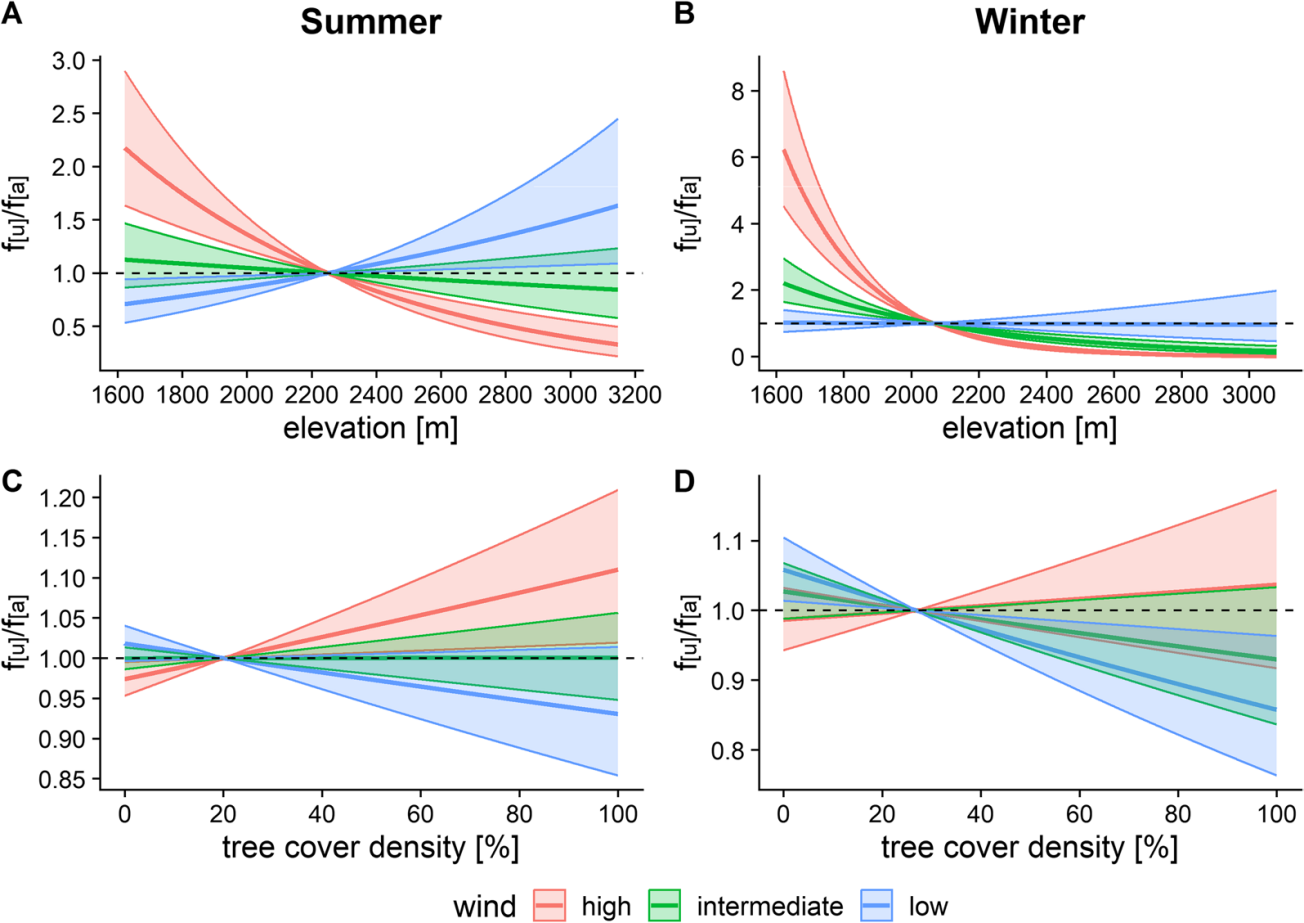
Effects of wind speed on chamois habitat choice with respect to elevation (**A**–**B**) and tree cover density (**C**–**D**) in summer (left panel) and winter (right panel). In the plots, low and intermediate wind speeds correspond to seasonal min and mean values, while maximum wind speed was set at 37 km/h (maximum = 95.4 km/h in the data) for summer, and 35 km/h (maximum = 88.9 km/h) for winter, determined visually from the density distribution of wind speed values. f_[u]_/f_[a]_ represents the frequency ratio between used and available positions, with values > 1 indicating preference and values < 1 avoidance. Note the different scales of the y-axis between graphs

With increasing wind speed, chamois moved to lower elevations in both summer and winter, but with a stronger selection in winter (Fig. 5A, B). Selection for denser tree cover occurred at high wind speeds in summer, while there was no preference at low or intermediate wind (Fig. 5C). In winter, on the other hand, selection occurred for open areas at low wind speeds with no preference at intermediate or high wind (Fig. 5D).

**Fig. 6:**
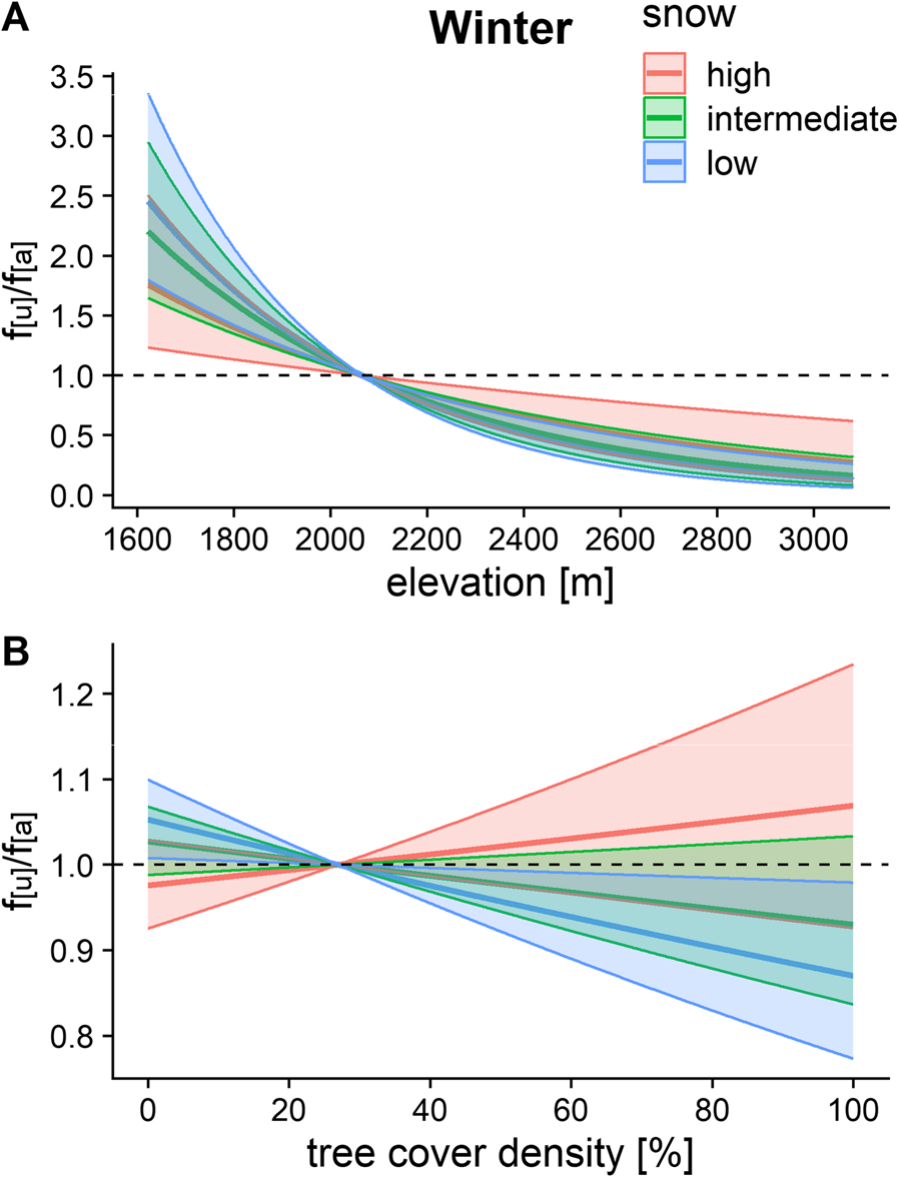
Effects of snow on chamois habitat choice with respect to elevation (**A**) and tree cover density (**B**) in winter. Low, intermediate and high snow correspond to minimum, mean and maximum values. f_[u]_/f_[a]_ represents the frequency ratio between used and available positions, with values > 1 indicating preference and values < 1 avoidance. Note the different scales of the y-axis between **A** and **B**

By comparison to the other weather variables, snow depth only had a weak effect on the habitat choice of chamois in winter. The animals preferred lower elevations, with only a marginally stronger selection at low snow cover (Fig. 6A). However, chamois preferred more open habitat at low snow cover (Fig. 6B).

**Fig. 7:**
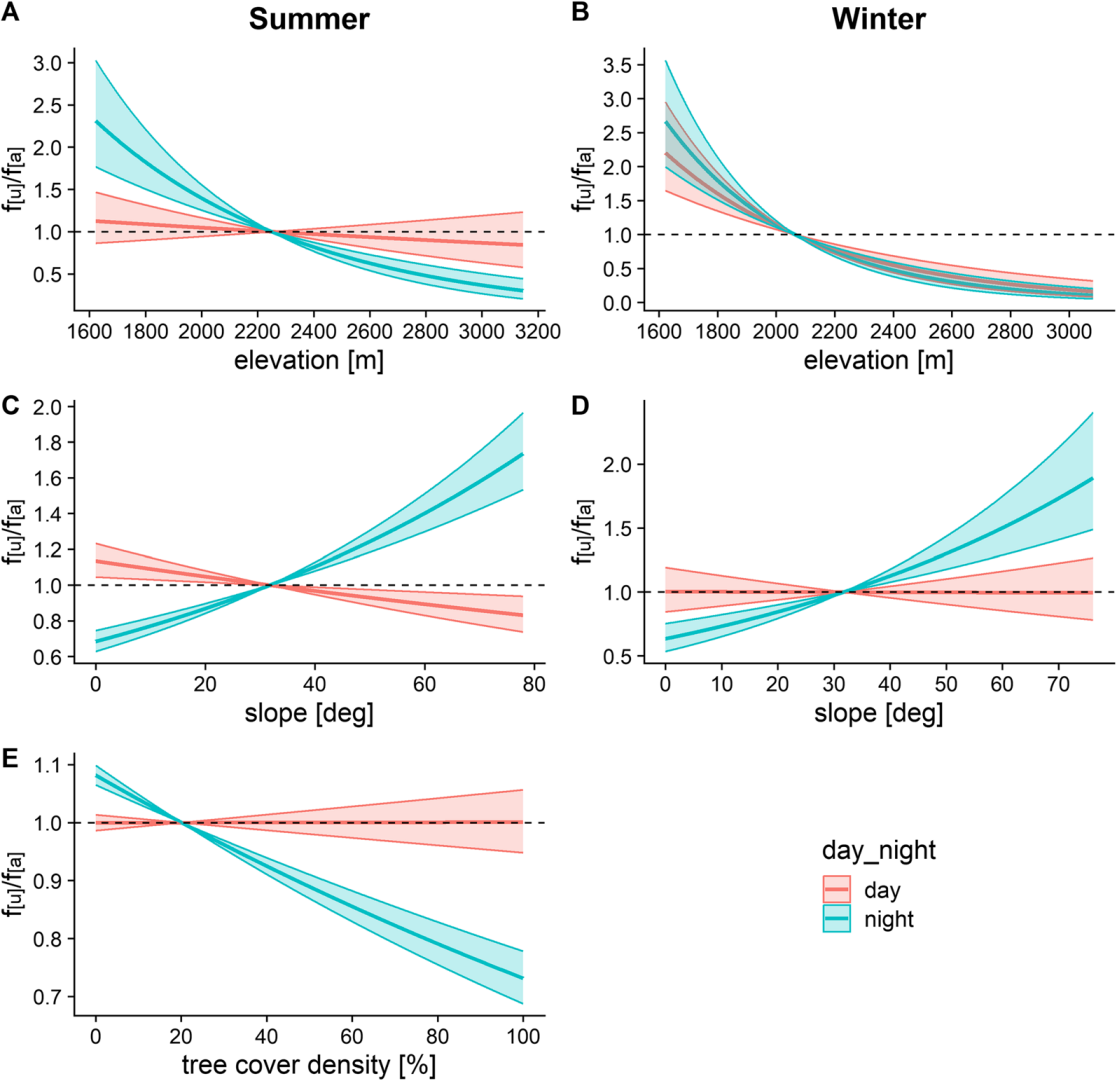
Effects of daytime on chamois habitat choice with respect to elevation (**A**–**B**), slope (**C**–**D**) and tree cover density (**E**) in summer (left panel) and winter (right panel). The interaction between daytime and tree cover density was not included in the final winter model according to AIC. f_[u]_/f_[a]_ represents the frequency ratio between used and available positions, with values > 1 indicating preference and values < 1 avoidance. Note the different scales of the y-axis between graphs

Regardless of daytime, the animals showed a preference for lower elevations in winter, with only a marginally stronger selection at night. This preference for low elevations at night persisted in summer, but there was no elevational selection during daylight hours at this time of year (Fig. 7A, B). A strong selection for steep slopes was detected at night during both summer and winter, while the animals showed a weak preference for shallower slopes during daylight hours in summer and no selection in winter (Fig. 7C, D). The interaction between daytime and tree cover density was included only in the final summer model, with no preference during daylight hours, but a strong selection for open habitats at night (Fig. 7E).

**Fig. 8:**
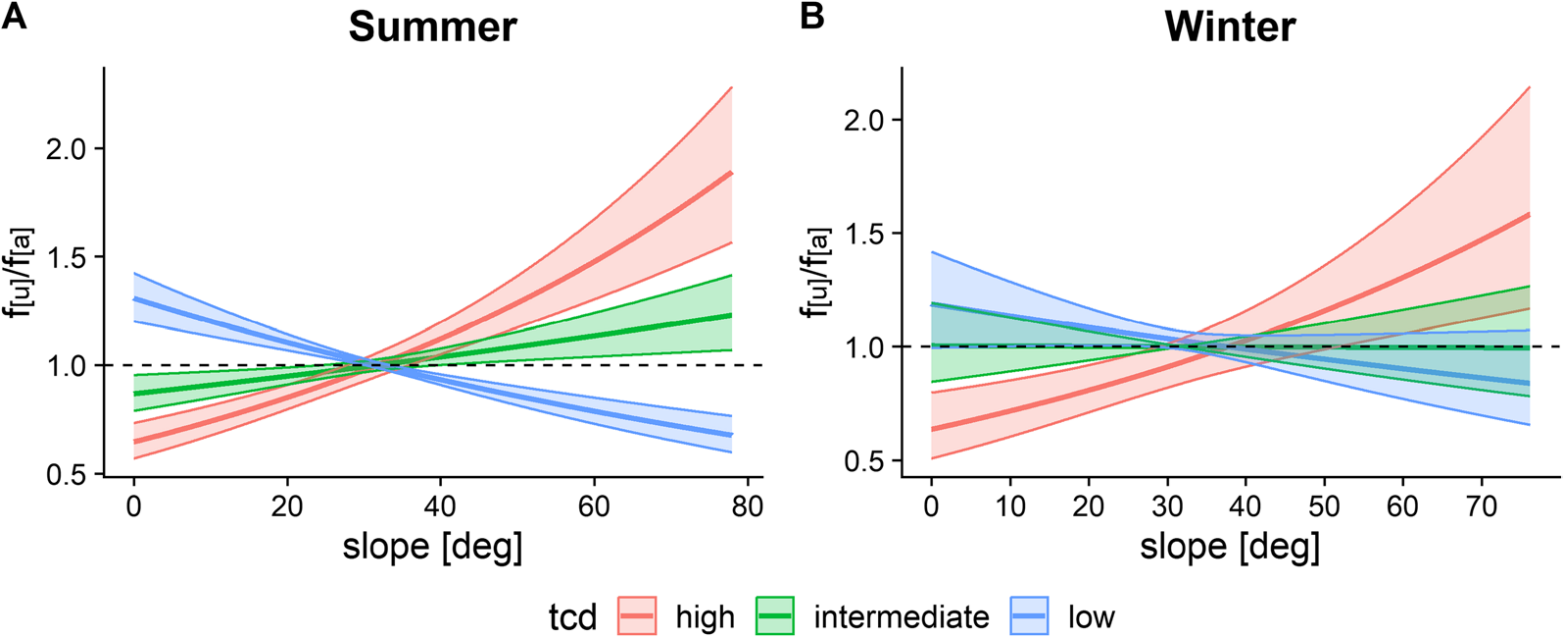
Effects of tree cover density (tcd) on the preference of different slopes by chamois in summer (**A**) and winter (**B**). Low, intermediate and high tree cover density correspond to minimum (i.e. completely open areas), mean and maximum values. f_[u]_/f_[a]_ represents the frequency ratio between used and available positions, with values > 1 indicating preference and values < 1 avoidance

Finally, there was a significant selection for tree cover density vs. slope, with chamois showing avoidance of steep slopes in open habitat, but a positive selection with increasing tree cover during summer (Fig. 8A). The relationship was similar in winter, but with a weaker selection in open habitat (Fig. 8B).

## Discussion

Using integrated step selection functions, we detected adaptive fine-scale adjustments of Alpine chamois habitat use in response to changing weather conditions in summer and winter. These included altitudinal movements, but also weather- and season-dependent use of different exposures and forest cover, and changes in potential escape terrain between day and night (Table 2).

**Table 2:** Expected relevance of explanatory variables/their first-order interaction terms for fine-scale habitat selection in Alpine chamois, and reason for their inclusion in the full models for summer and winter, respectively

Explanatory variable	Reason for inclusion	Model: relevant for chamois?
Eastness	Thermoregulation: earlier sunrise on easterly slopes to warm up after cold winter nights [68, 69]	Yes
Precipitation:elevation	Shelter: animals are more exposed to extreme weather conditions at high elevations	Yes
Precipitation:slope	Safety: (a) steep slopes as refuge from predators at times of poor visual and acoustic detectability in rainy conditions. On the other hand: (b) higher danger of slipping in steep terrain when wet	No (summer); yes (winter) No
Precipitation:tcd	Shelter: trees provide some protection from precipitation [26, 30]	No (summer); yes (winter)
Temperature:elevation	Thermoregulation: cooler temperatures with increasing elevation and exposure to wind along ridges [10, 22, 41]	No
Temperature:tcd	Thermoregulation: shade during high temperatures and some protection from cold temperatures by trees [17, 30, 74]	Yes
Temperature:northness	Thermoregulation: northerly slopes provide cooler habitat than southerly slopes [10]	Yes
Wind:elevation	Shelter: less exposure to wind at low elevations	Yes
Wind:slope	Safety: steep slopes as refuge from predators at times of poor acoustic detectability in windy conditions. On the other hand: higher danger of being blown off cliffs in high winds	No
Wind:tcd	Shelter: tree cover provides shelter from high winds [26, 30]	Yes
Snow:elevation	Safety/Mobility: high snow accumulation (drifts) at higher elevations decreases mobility of the animals	No
Snow:slope	Safety/Mobility/Foraging: steep slopes as safety habitat during times of decreased mobility in deep snow. Moreover, snow slides off steep slopes more easily and thus increases feeding opportunities [46]	No
Snow:tcd	Shelter/MobilityForaging: less snow accumulation under trees than in open habitat, and thus increased mobility and foraging opportunities [30]	Yes
Day/night:elevation	Thermoregulation: high elevations provide cooler conditions during the day, but animals may seek warmer temperatures at lower elevations during cool summer nights	Yes
Day/night:slope	Safety: steep slopes as safety habitat during darkness	Yes
Day/night:tcd	Thermoregulation/Shelter: forest attenuates temperature and weather extremes, so provides some protection during cold nights	Yes (summer); no (winter)
tcd:slope	Safety: steep slopes as safety habitat due to poorer visibility in the forest compared to open areas	Yes

The column ‘Model: relevant for chamois?’ indicates whether the variable/interaction showed the expected biological role according to the model results. tcd = tree cover density

### Thermoregulation

Important energy saving strategies in ungulates exposed to cold winter conditions consist in size reductions of their visceral organs [7], and the ability to decrease their heart rate and body temperature, thus lowering their thermoneutral zone and reducing energetic costs for endogenous heat production [6, 8, 9]. Chamois in the SNP showed a preference for southerly and easterly slopes in winter (Figs. 2B, 4F), and these preferences can be linked to both foraging opportunies and thermoregulation. Although the need for food intake is reduced in winter, some feeding on roughage is still essential, and southern slopes typically have lower snow pack than northern slopes, providing more accessible forage (e.g. [62]). However, the thermoregulation aspect may be even more important: Signer et al. [68, 69] showed that Alpine ibex, similarly to other mammalian taxa such as shrews [54], rodents [66], hyraxes [20] and primates [39], employ passive rewarming by basking in sunny areas after cold winter nights as part of their over-wintering strategy. The same authors also found that locomotor activity before rewarming was low, suggesting that ibex must have been close to these sunny areas already before sunrise. In winter, the first rays of sunlight appear in the southeast. If chamois employ similar over-wintering strategies as ibex, this would explain their simultaneous preference for southness and eastness. On southeasterly slopes, they could take advantage of the first rays of sunshine for warming up in early morning, while southern slopes enable them to remain in the sun during most of the daylight hours. Indeed, our results showed a stronger selection for southness than for eastness in winter (Table 1, Figs. 2B, 4F).

In summer, habitat selection by chamois for northerly vs. southerly slopes was dependent on temperature, with northerly slopes preferred at high and southerly slopes at low temperatures (Fig. 4E). As night-time temperatures in alpine environments are commonly around freezing even between June and October, a selection for southerly slopes at low temperatures also makes sense in summer, whereas northerly slopes with longer periods of shade were preferred at high summer temperatures (Fig. 4E). Chamois also responded to warmer temperatures in summer by moving to areas of denser tree cover (Fig. 4C), while there was only a tendency to move to higher elevations, associated with a large standard error (Fig. 4A). This strategy appears somewhat surprising, given that ibex prefer higher elevations at high summer temperatures [10, 41]. Active selection of forests at high temperatures is more consistent with findings for species living at lower elevations [17, 74], and may entail fitness trade-offs between thermoregulation and foraging needs, as forage quality and quantity is often reduced in the forest compared to open areas [5, 43, 64]. Chamois can be divided into two ecotypes—forest chamois and ridge chamois [14, 16], both of which co-occur in the SNP and are represented in our sample (Fig. 1). Interestingly, forest chamois in the SNP have smaller home ranges than ridge chamois [65], suggesting that they can satisfy their energetic requirements at a more local scale than ridge chamois. Indeed, Reiner et al. [58, 59] found that while chamois living in areas with higher proportions of forest cover in Austria were generally lighter, they were also less affected by widespread temporal declines in body mass with increasing temperatures observed in areas with little forest cover. This confirms that forests can indeed act as effective thermal buffers and would also agree with results from glucocorticoid analyses in the SNP: while chamois on meadows surrounded by forest responded to drought conditions with elevated stress levels, such a reaction was absent with respect to high summer temperatures [4], possibly because the animals regularly found shelter from the heat of the day in the forest. Forest chamois likely profit not only directly from more constant climatic conditions, but also the resulting increase in predictability of forage availability in the forest [55]. This may compensate for—and with climate change increasingly outweigh—the seasonally better, but in time and space more variable, forage availability in open areas.

Altitudinal selection became more relevant at low temperatures during summer, and at intermediate to high temperatures in winter, when chamois increasingly preferred lower elevations (Fig. 4B). While this pattern could also be explained by thermoregulatory needs in summer, it is contrary to expectations in winter. As the light summer coat of chamois provides limited protection against heat loss, and since temperatures typically increase with decreasing elevation, it is likely that chamois selected lower elevations in cold conditions for temperatures closer to their thermoneutral zone during summer. However, such relocation to lower elevations at high temperatures in winter would make no sense with respect to thermoregulation. A possible reason could instead be a safety aspect: increased risks of avalanches at higher winter temperatures make high elevations more dangerous, so that chamois retreat to lower elevations to avoid accidents. However, this would imply that the animals can gauge avalanche risks which to our knowledge has not been shown to date.

### Shelter

Responses to rain or snowfall were restricted to high levels of precipitation and dominated by altitudinal movements in both summer and winter (Figs. 3A, B). The latter also applied to wind speed (Figs. 5A, B). In summer, strong precipitation and high wind speeds are often associated with thunderstorms; in winter, heavy snowfall increases the danger of avalanches. Both represent conditions under which a retreat to lower elevations would also be adaptive from a safety perspective. As forest cover only played a secondary role in the reaction to both wind and precipitation (with large standard errors; Figs. 3D, 5C & D), it is unclear whether seeking shelter was the main driver for the pronounced downward movement of the animals, or if avoidance of environmental risks (avalanches, landslides or thunderstorms) during inclement weather conditions at high elevations might also have played a role.

The weak effects of snow depth on altitudinal (Fig. 6A) and tree cover (Fig. 6B) selection could be explained by fine-scale heterogeneity in snow depth depending on topography, snow drift etc. that can vary within the same elevations or habitat type. On the other hand, in contrast to other weather variables, snow cover (even if not depth) in winter is predictable. Chamois therefore appear to react to the first snow relatively early by moving to wintering ranges that are typically located at lower elevations and/or in forested areas regardless of snow depth (see also [46]). Within these wintering ranges, the animals then reduce their activity levels in response to increasing snow depth [18], which represents an energetically more efficient behaviour than trying to relocate in deep snow.

### Safety

Selection patterns of steep slopes may be complex to explain for mountain ungulates: on the one hand, they represent important escape terrain from predators [12, 63, 77] and provide foraging areas in winter where the snow slides off quickly so that vegetation beneath it is easier to reach (e.g. [46]). On the other hand, they also represent dangerous terrain with respect to avalanches and the possibility of falling off cliffs, particularly in icy conditions. Our results suggest that the role of steep slopes as escape terrain where the animals feel safe from potential predators outweighs all other aspects. Chamois selected steeper slopes during periods of strong snowfall (though only marginally, Fig. 3C), with increasing tree cover density (while flatter areas were preferred in open terrain in summer; Fig. 8) and particularly at night (Fig. 7C, D), i.e. under conditions of low visual detectability of potential predators. On the other hand, the interaction terms of slope with wind (increased danger of falls on steep slopes in strong winds) or snow depth (better forage conditions, but increased danger of avalanches on steep slopes) were not included in the final models.

Chamois altered their habitat preferences not only in response to weather conditions, but also between day and night: compared to daytime hours, they selected for lower elevations, but avoided forest cover at night in summer (Fig. 7A, B, E). Together with the strong preference for steep slopes at night during both summer and winter (Fig. 7C, D), these diel movement patterns most likely represent a combined selection for escape terrain (steep but open habitat) and protection from thermal exposure (low elevations due to colder nights at higher altitudes). Diel migrations have previously been reported in chamois [29], but in the context of human disturbance. In their study, the animals moved closer to hiking trails at night, but avoided them during daytime. As distance to hiking trails is strongly correlated with elevation in the SNP (there are few high elevation trails), we cannot completely rule out the possibility of diel movement patterns with respect to elevation also being related to the presence of visitors on trails during daytime in summer. However, the effect persisted in winter (albeit less pronounced, as chamois spend the winter at lower elevations anyway), when the SNP is closed to visitors and human disturbance can therefore be ruled out. This suggests a more important role of lower elevations as thermal shelter at night.

### Critical chamois habitat under climate change

Due to summer visitors being restricted to hiking on designated trails only and the closure of the park to visitors in winter, the changes in habitat use according to weather conditions observed here can be assumed to be largely independent of anthropogenic influences. We found support for thermoregulation, shelter and safety from predators all being relevant in the habitat choice of chamois. The important role of escape terrain in the form of steep slopes suggests that the long absence of mammalian predators from the area has not reduced the animals’ strong preference for refuge areas where they feel safe from predation, particularly under conditions of poor visibility. While forest cover seemed less crucial as shelter from precipitation or wind than elevation, it played an important role as thermal refuge from high summer temperatures. By contrast to Alpine ibex, which seek high elevations on hot summer days and reduce their food intake during this time [10], a strategy of retreating to the forest at high temperatures may not necessarily need to involve trade-offs between thermoregulation and energy balance. This is demonstrated by the smaller home ranges of ‘forest chamois’ [65] along with better ability of animals to maintain their body weights with increasing temperatures in areas where forest is widely available [58, 59]. Critical habitat for chamois under climate change and with the return of large mammalian carnivores will thus involve subalpine forests as thermal refuges with steep cliffs as escape terrain. Some known important ungulate wintering areas in the Swiss Alps and foothills are already protected from human disturbance as ‘wildlife quiet zones’, where human entry is prohibited in winter, so as not to disturb wildlife at this crucial time [61]. Future management implications may need to include setting aside critical habitats for the animals in summer and protecting them from human disturbance similarly as in winter.

## Conclusions

Although various mountain ungulate species increasingly move upslope with warmer summer temperatures in the wake of climate change [22], fine-scale responses in habitat use to changing weather conditions are more complex. Specifically, forests not only provide shelter from inclement weather conditions for Alpine chamois, but also important thermal refuges during summer (see also [58, 59]). The role of these refuge areas should not be underestimated, and their access to the animals ensured at all times by protecting them from human disturbance [67].

### Supplementary Information

**Additional file 1. Figure A1:** Environmental variables for the study area. **Table A1:** Pearson’s correlation coefficients between explanatory variables.

## Data Availability

The datasets used and analysed during the current study are available from the corresponding author on reasonable request.
