# Supplementary material for: Weather-dependent changes in habitat use by Alpine chamois
Source: Mov Ecol. 2024 Jan 16;12:3. doi: 10.1186/s40462-024-00449-x (PMC10792825; doi:10.1186/s40462-024-00449-x)
Supplement: Supplementary file 1 — Additional file 1. Figure A1: Environmental variables for the study area. Table A1: Pearson’s correlation coefficients between explanatory variables. [file 40462_2024_449_MOESM1_ESM.docx]

**Appendix**

| 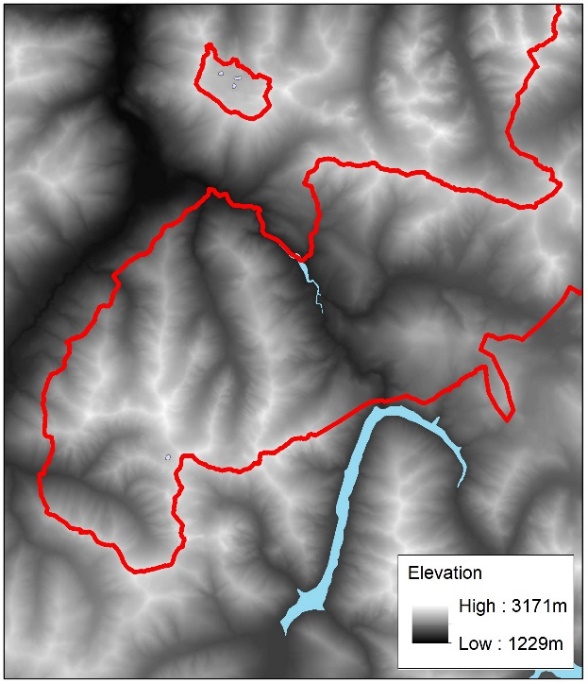 | 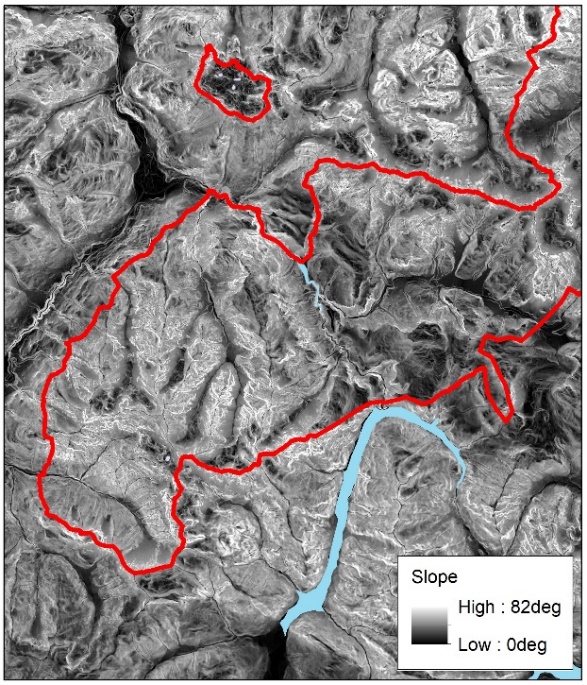 |
| --- | --- |
| 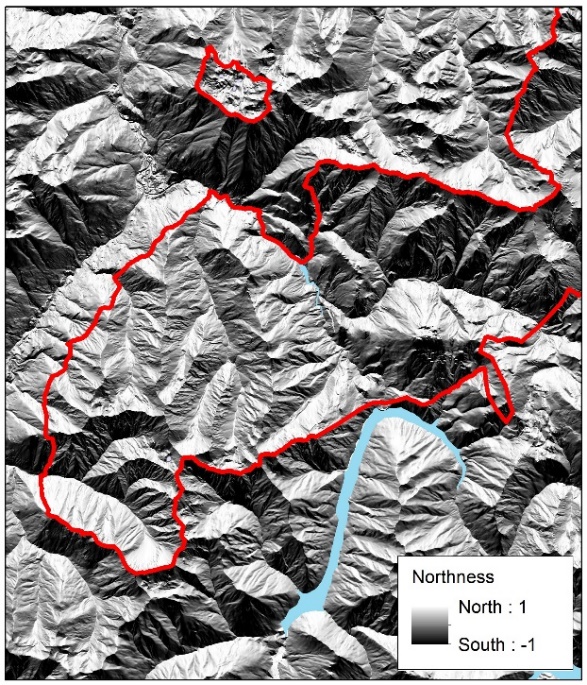 | 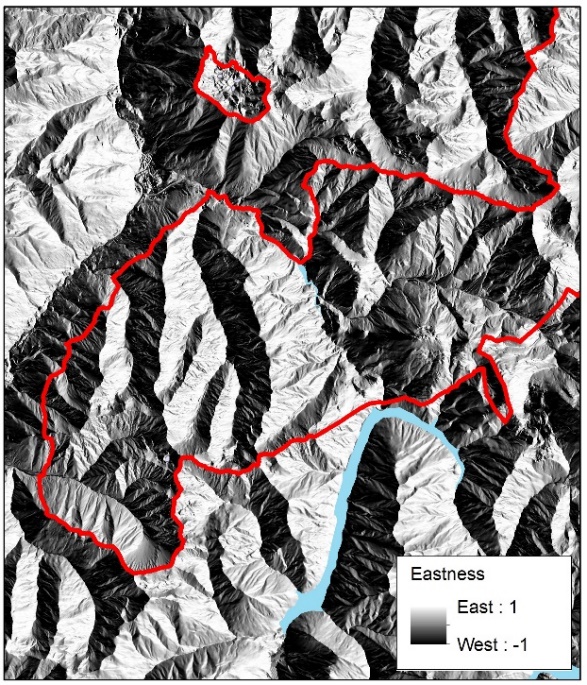 |
| 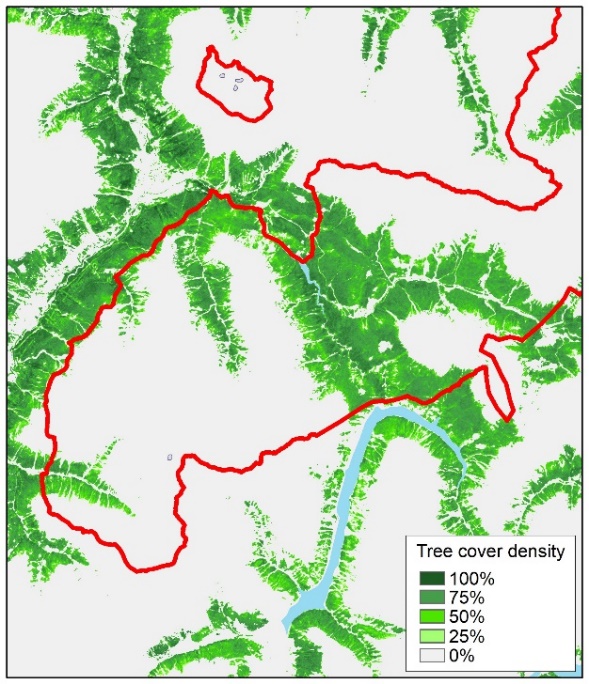 | Figure A1. Environmental variables for  the study area used in the models for chamois habitat use. The red line  represents the boundary of the Swiss National Park, light blue areas  represent lakes. |

Table A1. Pearson’s correlation coefficients between explanatory variables for the summer (upper right diagonal) and winter (lower left diagonal) model. As p-values in correlations strongly depend on sample sizes, only the coefficients without p-values are indicated here.

|  | step length | elevation | slope | tcd | north-ness | east-  ness | precipitation | temperature | wind |
| --- | --- | --- | --- | --- | --- | --- | --- | --- | --- |
| step length | - | 0.100 | -0.015 | -0.016 | 0.055 | 0.036 | 0.002 | 0.025 | 0.015 |
| elevation | 0.124 | - | 0.211 | -0.616 | 0.153 | 0.232 | -0.017 | 0.111 | 0.021 |
| slope | -0.055 | 0.268 | - | -0.255 | -0.033 | 0.044 | -0.003 | -0.008 | -0.024 |
| tcd | 0.019 | -0.469 | -0.253 | - | -0.009 | -0.240 | -0.001 | -0.033 | 0.007 |
| northness | 0.121 | 0.008 | -0.078 | 0.133 | - | 0.015 | -0.004 | 0.055 | 0.008 |
| eastness | -0.021 | 0.139 | 0.149 | -0.148 | -0.014 | - | 0.015 | 0.001 | -0.008 |
| precipitation | -0.002 | -0.038 | -0.011 | 0.018 | 0.008 | 0.017 | - | -0.064 | 0.011 |
| temperature | 0.014 | 0.032 | -0.014 | -0.042 | <0.001 | -0.003 | 0.073 | - | 0.481 |
| wind | 0.011 | -0.037 | -0.017 | 0.010 | -0.003 | 0.005 | 0.102 | 0.403 | - |
| snow | 0.008 | -0.093 | 0.033 | 0.031 | -0.089 | 0.068 | 0.018 | -0.214 | 0.040 |
